# Employing in silico investigations to determine the cross-kingdom approach for *Curcuma longa* miRNAs and their human targets

**DOI:** 10.1186/s43088-022-00330-z

**Published:** 2023-01-07

**Authors:** Atiyabanu N. Saiyed, Abhay R. Vasavada, S. R. Kaid Johar

**Affiliations:** 1 Department of Cell and Molecular Biology, Iladevi Cataract and IOL Research Centre, Ahmedabad, Gujarat India; 2 Ph.D. Scholar of Manipal Academy of Higher Education, Manipal, Karnataka India; 3 Department of Zoology, BMTC, Human Genetics, USSC, Gujarat University, Ahmedabad, Gujarat India

**Keywords:** Plant elements, Phytochemicals, *Curcuma Longa*, miRNAs, Cross-kingdom

## Abstract

**Background:**

Plant elements and extracts have been used for centuries to treat a wide range of diseases, from cancer to modern lifestyle ailments like viral infections. These plant-based miRNAs have the capacity to control physiological and pathological conditions in both humans and animals, and they might be helpful in the detection and treatment of a variety of diseases. The present study investigates the miRNA of the well-known spice *Curcuma Longa* and its prospective targets using a variety of bioinformatics techniques.

**Results:**

Using the integrative database of animal, plant, and viral microRNAs known as miRNEST 2.0, nine *C. longa* miRNAs were predicted. psRNA target service foretells the presence of 23 human target genes linked to a variety of disorders. By interacting with a variety of cellular and metabolic processes, miRNAs 167, 1525, and 756 have been found to be critical regulators of tumour microenvironment. SARS-cov2 and influenza A virus regulation have been connected to ZFP36L1 from miRNA 1525 and ETV5 from miRNA 756, respectively.

**Conclusions:**

The current cross-kingdom study offers fresh knowledge about how to increase the effectiveness of plant-based therapies for disease prevention and serves as a platform for *in vitro* and *in vivo* research development.

**Graphical abstract:**

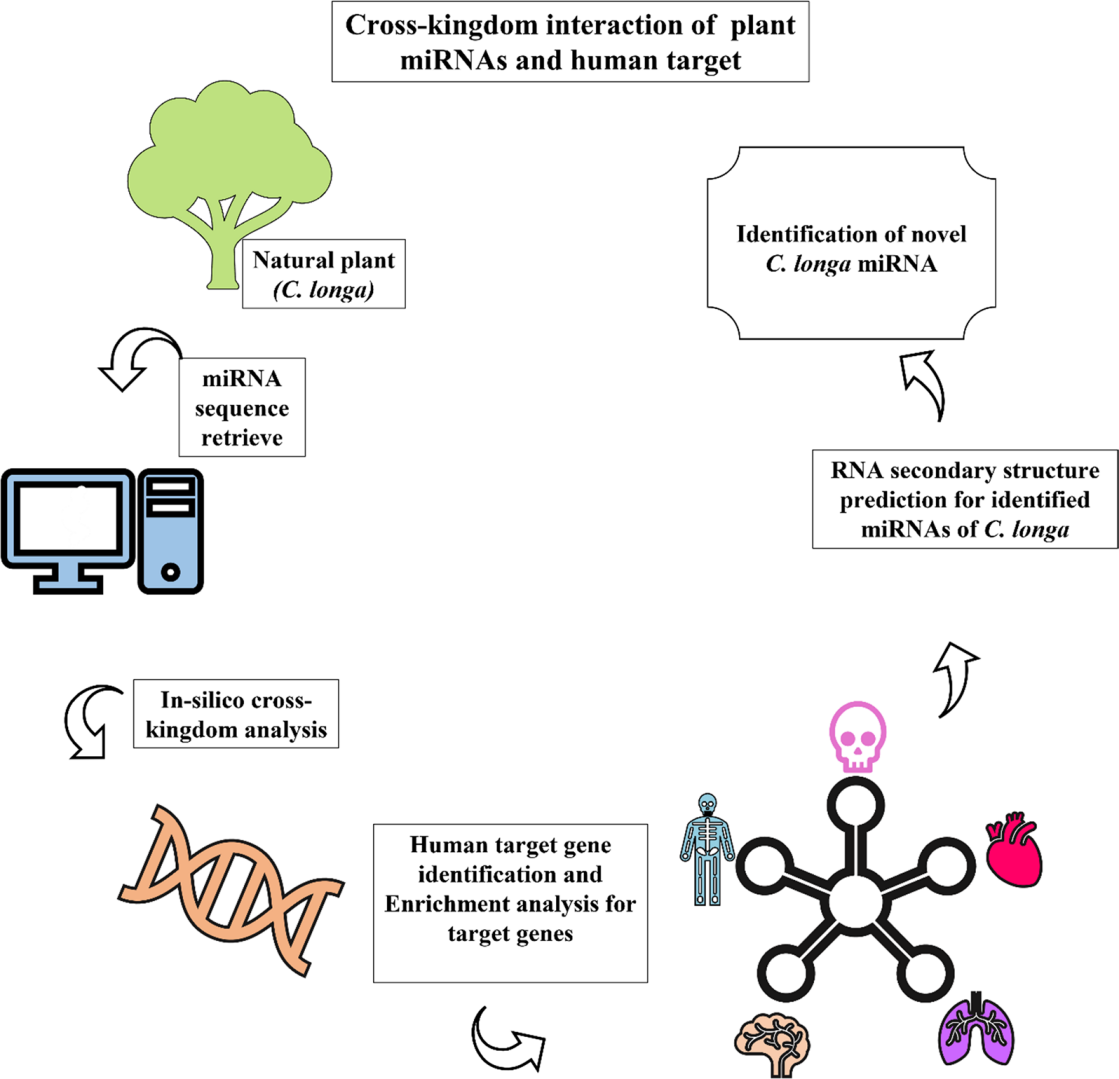

## Background

In this revolutionary chorology, the appropriateness of plant natural products has a fructifying tendency in enhancing mankind's robustness by manifesting plants, plant parts, and plant products into daily life as active intake and dietary supplements. Bioprospecting identifies more significant and abundant plant-based compounds with pharmacological value. Modern herbal compounds such as saponins, alkaloids, and flavonoids may also be able to regulate the mechanisms of Severe Acute Respiratory Syndrome Coronavirus-2 (SARS-CoV-2) by inhibiting their main protease (Mpro) enzymes [1]. Because living plant species contribute more to the world's chemical diversity of bioactive compounds than any man-made synthetic library, finding novel plant molecules today would necessitate more advanced and powerful discovery perspectives. *Curcuma Longa* (*C. longa*), a plant belonging to the *Zingiberaceae* family, has been used for medicinal purposes since ancient times. Powder form of dry roots of *C. longa* is referred as turmeric and it is most common spice in various cuisines across the world. Medically turmeric has been used to treat gastrointestinal problems, biliary and hepatic disorders, diabetic sores, rheumatism, inflammation, sinusitis, anorexia, coryza, and cough. It has anti-cancer, anti-diabetic, antioxidant, hypolipidemic, anti-inflammatory, antibacterial, anti-fertility, anti-venom, hepatoprotective, nephroprotective, anticoagulant, and anti-HIV properties [2, 3]. The purpose of this study is to discover the potential impact of *C. longa* miRNAs on human target genes and SARS-CoV2 infection. This study differs from prior studies on *C. longa* miRNAs in terms of data identification, cross-kingdom interaction with human genes, and disease regulation pathways.

### The achievements of plant miRNAs on human health

RNA interference (RNAi) is the most significant scientific achievement in the last two decades, and it is currently being used in clinical studies. With recently identified new class of RNAi molecules, microRNA (miRNA) is now playing an even well-known role in research and technology [4]. miRNAs are the most important genomic regulators, controlling 1–4% of all human genes. Small non-coding RNAs that have 21–25 nucleotides and are classified as miRNAs. miRNAs modulates mRNA content at the post-transcriptional stage to aid many important activities in biological systems, including embryonic development, cell maintenance, chemical signalling, and cell apoptosis. An anomaly in miRNA expression has been linked to the onset of a number of diseases ranging from viral to various cancers. miRNA can attach to viral RNA and inhibit viral genome processes by binding to the open reading frame (ORF) and slowing down the translation process. miRNAs have the ability to regulate cells in an autocrine, paracrine, and endocrine manner [5]. As a result, miRNAs has emerged as a possible marker for evaluating and diagnosing disease development [6]. miRNA continues to explore its path towards the prevention and treatment of human diseases. As an application, plant miRNAs could be used in the research and treatment of a variety of disorders. There are cutting-edge researches specifically demonstrating cross-kingdom gene transfer, mechanism of plant miRNAs absorbance, metabolism, and distribution on target site. Plant miRNAs absorption causes the intestine's epithelial cells to incorporate with miRNAs via a variety of processes, allowing plant miRNAs to reach gut cells, be delivered to specific physiological sub-compartments, and modulate gene expression in various body systems [7]. The first evidence of cross-kingdom gene transfer was proclaimed by Zhang et al. [8] plant miRNA 168a have ability to regulate LDLRAP1 gene in mice model and found significant decrease in LDLRAP1 and increase level of miRNA 168a illustration that intake of plant miRNA can instantaneously alternate the LDL gene expression. Moreover, the miRNAs from strawberry perhaps customized the toll-like receptor adherence capacity that corresponds to autoimmune response and dendritic cell migration. G-protein subunit alpha 12 (GNA12) involved regulation m-TOR signalling cascade, a synthetic isoform of plant miRNA 171 suppress the activity of this compound in human embryo kidney cells (HEK293) and affecting m-TOR mechanism [9]. However, plant miRNAs mechanisms are differed from animal miRNAs because of its methyl and 2-hydroxyl group (2-OH) at 3’ terminus. These groups improve the integrity and stability of plant miRNA and hydroxyl group slower down the degradation rate in synthetic plant miRNAs [10]. The recent accumulating attestation on horizontal gene transfer indicates although, these evidences have criticism and scepticism concerning the dependability and sensitivity of the techniques applied for cross-kingdom miRNAs transmission. Cross-kingdom regulation of plant-derived miRNAs needs attention because of its potential to create novel therapeutic treatments for miRNA deregulation-related illnesses.

### Computational approaches for studying miRNAs and its effects on disease prevention across the kingdoms

Plant miRNAs have a significant impact on horizontal gene transfer mechanisms and may become an important research topic to investigate. In terms of plant miRNAs' ability to regulate gene expression across kingdoms, very few studies have used in silico approaches to assess their role in human disease targets and disease regulation. When developing plant-based miRNAs for mammalian genes, there are four primary properties that are often exploited. These include base-pairing between the 'seed' region and the target gene, low free energy estimation (genuine paring with miRNA target), target prediction (possible binding sites necessary for cross-kingdom transfer) and site accessibility [7]. These principles are well-founded for predicting plant-based miRNAs transfer in mammalian genes. We have been working on bioinformatics for a long time and have a large collection of cross-kingdom systematic results. In our *in silico* research, we discovered that the miRNAs of *Ocimum basilicum* [11], *Bacopa monnieri* [12], *Persea americana* [13], and *Prunus armeniaca* [14] all might play a direct role in disease state and maintenance in our *in silico* research. The *C. longa* miRNAs are not been evaluated as well their human target genes are not known. Therefore, in the present study, we have identified miRNA of *C. longa* from various data sources and their human target genes are identified. Through this study, we would like to emphasize that some effects of turmeric may be mediated by its miRNAs.

## Methods

**Fig. 1:**
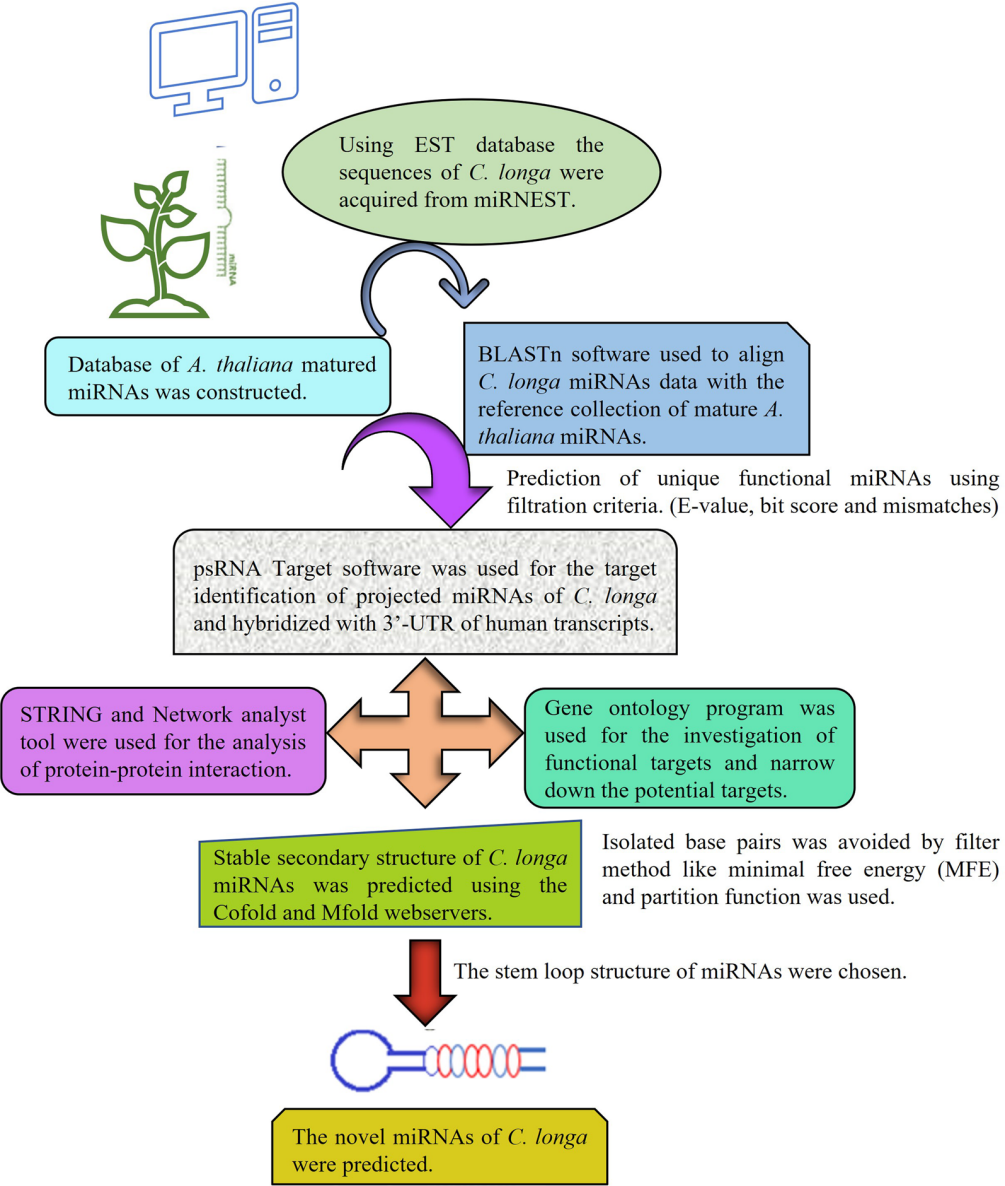
A structural flow chart of methods

*C. longa* miRNA sequences were retrieved from the miRNEST 2.0 sequence prediction database (http://mirnest.amu.edu.pl). *Arabidopsis thaliana* (*A. thaliana*) mature miRNAs was used to set reference miRNAs for prediction. The first step was to create a database of matured A. thaliana miRNAs. *C. longa* ESTs were aligned with a reference sample of mature *A. thaliana* miRNAs using BLASTn software [15]. Following alignment, filtration criteria were used to extract unique miRNAs for predicting functional miRNAs (E-value, bit score, and mismatches, among others). Target human gene interactions were carried out using the psRNATarget software, and these projected miRNAs were then hybridized with the 3'-UTR of human transcripts [16]. Gene enrichment ontology analysis of functions of targets was investigated using the ShinyGO software. This is done in order to limit down possible targets for further investigation [17]. STRING and network analyst computational software were utilized to determine human gene–gene and gene-protein interaction for these discovered miRNA targets [18–21]. The bottleneck, stress, and betweenness algorithms were used to assess the target genes in Cytoscape v 3.9.1 cytohubba plugin [22]. Utilizing the Cofold web server, filters like the minimal free energy (MFE) and partition function are applied to prevent isolated base pairs and concentrated parameters. The most stable secondary structure of RNA can be predicted using this web server [23, 24]. The only stem loop structure of miRNAs was chosen for the study of miRNAs of *C. longa*. Because the stem loop structure is crucial for the secondary RNA structure because it gives miRNAs their structural integrity. Furthermore, stem loop topology may have an impact on enzymatic activities and aids in the provision of recognition sites for RNA binding proteins [25]. The purpose of this study is to evaluate the miRNAs that have been anticipated [26, 27]. A structural flow chart of methods is depicted in Fig. 1.

## Results

### *C. longa* sequence prediction using miRNEST 2.0

miRNEST analysis provide the identification of miRNAs in plants animals and viruses. Using an EST database, this software discovered miRNAs in plants and animals. This application has identified a total of 10,004 miRNAs in 199 plant and 221 animal kingdoms. miRNEST 2.0 is an improved version that recognizes a total of 39,122 miRNAs [15]. The most recent edition of miRBase (version 22) has information on 38,589 pre-miRNAs from 271 organisms, including 1917 precursors and 2654 mature miRNAs in humans. It provides data for 326 hairpins and 428 mature sequences for A. thaliana and 258 hairpins and 469 mature sequences for Drosophila melanogaster as examples of other model species [28]. In the present study, we used this software to identify, predict and retrieve the sequences of *C. longa* miRNAs. The predicted miRNAs are listed in Table 1. Furthermore, these miRNAs were also tested using the miRBase in silico database. A total of 9 miRNAs were identified for further evaluation (Table 1).

**Table 1:** List of predicted *C. longa* miRNAs using miRNEST 2.0 database

No	miRNEST id	Species	Family	Source	Mature miRNA	vs miRBase
1	MNEST000500	Curcuma longa	MIR1525	miRNEST	TGAGTTAATTAAGTTTTTATG	NO HITS
2	MNEST000501	Curcuma longa	MIRf129	miRNEST	TCCGGAGGGATCCCTTCCTTG	NO HITS
3	MNEST000502	Curcuma longa	MIR167	miRNEST	TGAAGCTGCCAGCATGATCTC	sbi-MIR167d: 0.00000004
4	MNEST000503	Curcuma longa	MIR167	miRNEST	TGAAGCTGCCAGCATGATCTG	sbi-MIR167c: 0.000000007
5	MNEST000504	Curcuma longa	MIRf756	miRNEST	AGATCATCTGGCAGTTTCAAT	sbi-MIR167c: 0.000000007
6	MNEST000505	Curcuma longa	MIRf1568	miRNEST	CGGCGTCGTCTTCGCTCCCGA	NO HITS
7	MNEST045832	Curcuma longa	miR167	microPC	AAGCTGCCAGCATGATCT	osa-MIR167h: 0.0005
8	MNEST044906	Curcuma longa	MIR167	microPC	TGAAGCTGCCAGCATGATCTC	osa-MIR167h: 0.00003
9	MNEST046454	Curcuma longa		microPC	TGAAGCTGCCAGCATGATCT	osa-MIR167h: 0.00003

### Analysis and interaction of human targets of *C. longa* miRNA

The psRNATarget tool is effective for validating miRNA–miRNAs interactions [16]. The psRNATarget software was developed to identify the target genes of predicted plant miRNAs. This software uses a complementary mismatch-sensitive 'seed' region to identify potential targets for interested miRNAs. This software estimates mRNA target availability and energy to unwind the secondary structure throughout the target site to calculate target accessibility. This software's performance, most importantly, estimates mRNA target availability. This technique was used to investigate the *C. longa* plant miRNAs target gene for humans, and a total of 23 target genes were discovered, all of which play a key role in modulating human diseased conditions. In a recent study, the target gene ZFP36L1 from miRNA 1525 also had a role in the regulation of the influenza A virus through translational repression [29]. miRNA 167 and miRNA 1525 significantly inhibit mitogen-activated protein kinases (MAPK) and NF-kB signalling pathways in cross-kingdom analysis, indicating that these two miRNAs are likely beneficial for maintaining cell proliferation and angiogenesis and play a role in regulating the tumour microenvironment. The miRNA 756 target gene ETV5 plays an important role in maintaining lung homoeostasis, and its activity has been found to be reduced in COVID-19 patients [30, 31] (Table 2).

**Table 2:** The target prediction and accessibility of *C. longa* miRNAs using the psRNATarget in silico tool

miRNAs	Sequence	Targets	Inhibition	Probable Function	miRNAsstart	miRNAs end	Targets start	Targets end	Target aligned fragment
miRNA167	TGAAGCTGCCAGCATGATCTC CUCUAGUACGACCGUCGAAGU	PPP3R2	Cleavage	Inhibit MAPK and GPCR pathway	1	21	1205	1225	UAUGUCAUGUUGGUAGCUUUA
miRNA1525	TGAGTTAATTAAGTTTTTATG GUAUUUUUGAAUUAAUUGAGU	TNFSF15	Cleavage	Activate both the NF-κB and MAPK signalling pathways Prevent apoptosis Inhibits cell proliferation and angiogenesis	1	21	4011	4031	UAUAAAAGUUUAACUAACUCA
		PCGF5	Cleavage		1	20	1953	1972	AUGGAAUUUUAAUUAACUCA
		RHD	Cleavage		1	21	689	709	CAUAGAAACUUAAUUAGAUUA
		YIPF6	Translation	Inhibits vesicle formation, trafficking, and budding	1	21	3017	3037	UAUAAAAACUCAAUUGAUUCC
		SETD7	Cleavage	Inhibit expression of collagenase and insulin gene Stabilizes p53/TP53 and increasing p53/TP53-mediated transcriptional activation	1	21	3614	3634	UAUAAAAAUUUAAAUCACUCA
		DCC	Cleavage	Inhibits apoptosis Reduces tumour suppressor activity	1	21	2914	2934	UAUAAAAAUUUAGAUAGUUCA
		ATRN	Cleavage	Prevent obesity Reduces inflammation	1	20	656	676	UAUAGUAACUUGAUUAAUUUA
		SLC25A42	Cleavage	Alters mitochondrial transport of proteins	1	20	1992	2011	AUAAAAACUUAAAUGACUUC
		CLEC2D	Cleavage	Prevent Osteoporosis type of condition	1	20	313	332	AAAGAGAUUUAAUUGACUCA
		SRXN1	Translation	Reduces resistance to oxidative stress Increases activity of anti-oxidative stress enzymes	1	20	2070	2089	AUAAAAGUUUAUUUAAUUUA
		ALPK1	Cleavage	Influence neuronal coordination	1	20	769	788	AAAGAGGUUUAAUUAACUCA
		ZFP36L1	Translation	Alters effect of growth factors and other cytokines	1	21	1151	1171	UAAAAAAGCUUAUUUAACUUA
		HDX	Translation	Alter activity of several transcription factor	1	20	458	477	UUAAAAAAUUAUUUAACUCA
		GAB1	Cleavage	Prevent tubulogenesis, cellular growth response, growth transformation and apoptosis	1	20	1945	1964	AUAAAUACAUGAUUAAUUCA
		PNPO	Cleavage	Prevent epileptic changes	1	20	688	707	AUUACAACUUAACUAACUCA
miRNA1568	CGGCGTCGTCTTCGCTCCCGA AGCCCUCGCUUCUGCUGCGGC	CD99L2	Cleavage	Prevent leucocyte infiltration and subsequent immune response	1	21	304	324	GCGGGGGCCAAGAUGAUGCUG
miRNA 129	TCCGGAGGGATCCCTTCCTTG GUUCCUUCCCUAGGGAGGCCU	PGM3	Cleavage	Prevents glycogen storage and utilization Prevent resistance to diabetic nephropathy and neuropathy	1	21	1901	1921	AAAGGAAGGGAUCCCUCAGGA
		NAPB	Translation	Prevent amyotrophy and hereditary neuralgic	1	21	600	620	UGAGGAAGGGAGCUUUCUGGA
		DLG2	Cleavage	Neurological role	1	21	483	503	AAAGAAAGGAAUCCCUUUGGA
miRNA 756	AGATCATCTGGCAGTTTCAAT UAACUUUGACGGUCUACUAGA	CYB5B	Cleavage	Prevent accumulation of oxygenases	1	21	639	659	CUUAAAACUGCCAAAUGAUUU
		ETV5	Cleavage	Prevent neurofibromatosis, type 2 and other neurological conditions	1	21	2206	2226	CUUGAACCUGCCAGCUGAUUU
		GREM2	Cleavage	Prevent metastasis and cell migration differentiation	1	21	3138	3159	UUUGAAAUUGGCCAGAUGAUUU

### Gene enrichment analysis for targeted genes 

**Fig. 2:**
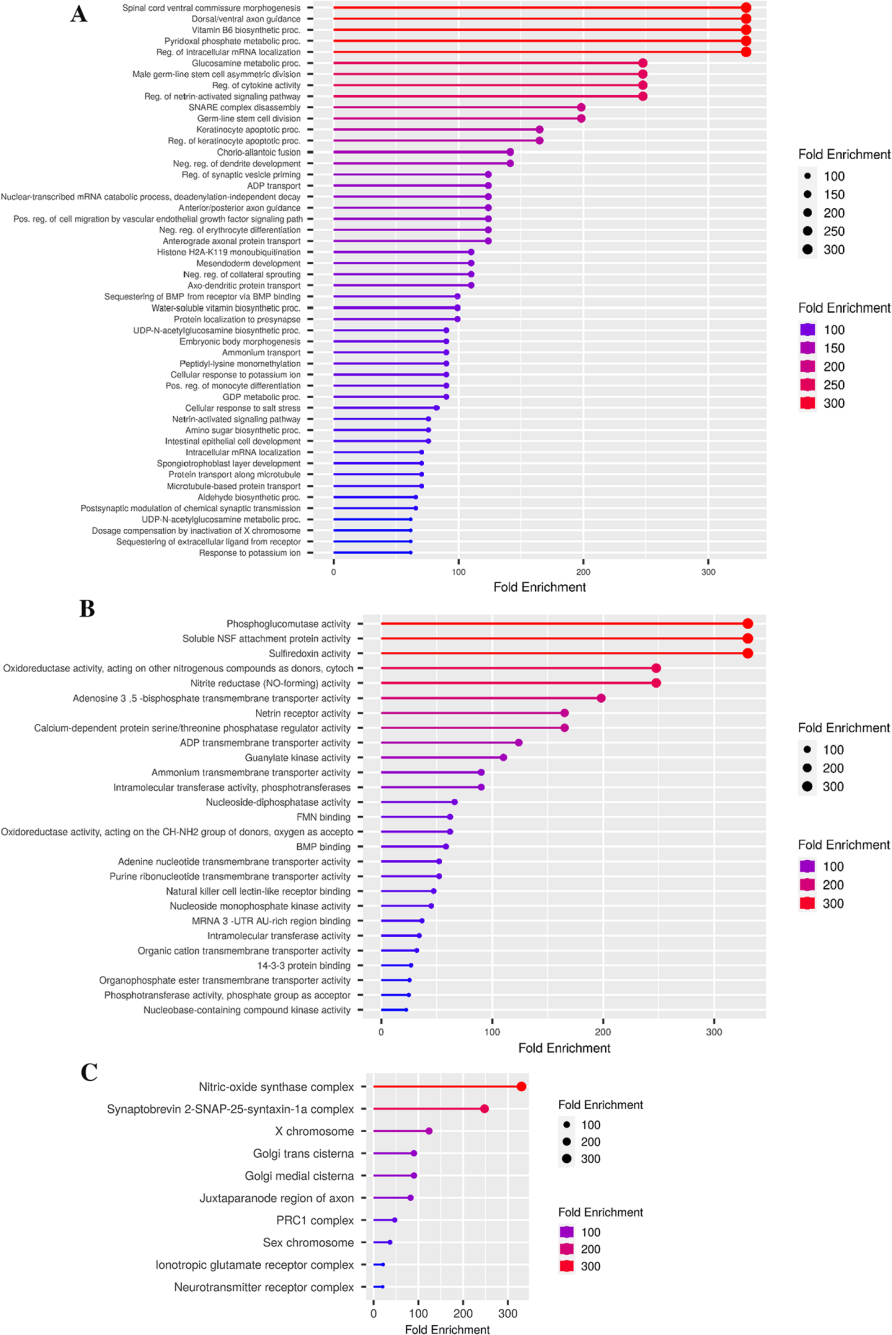

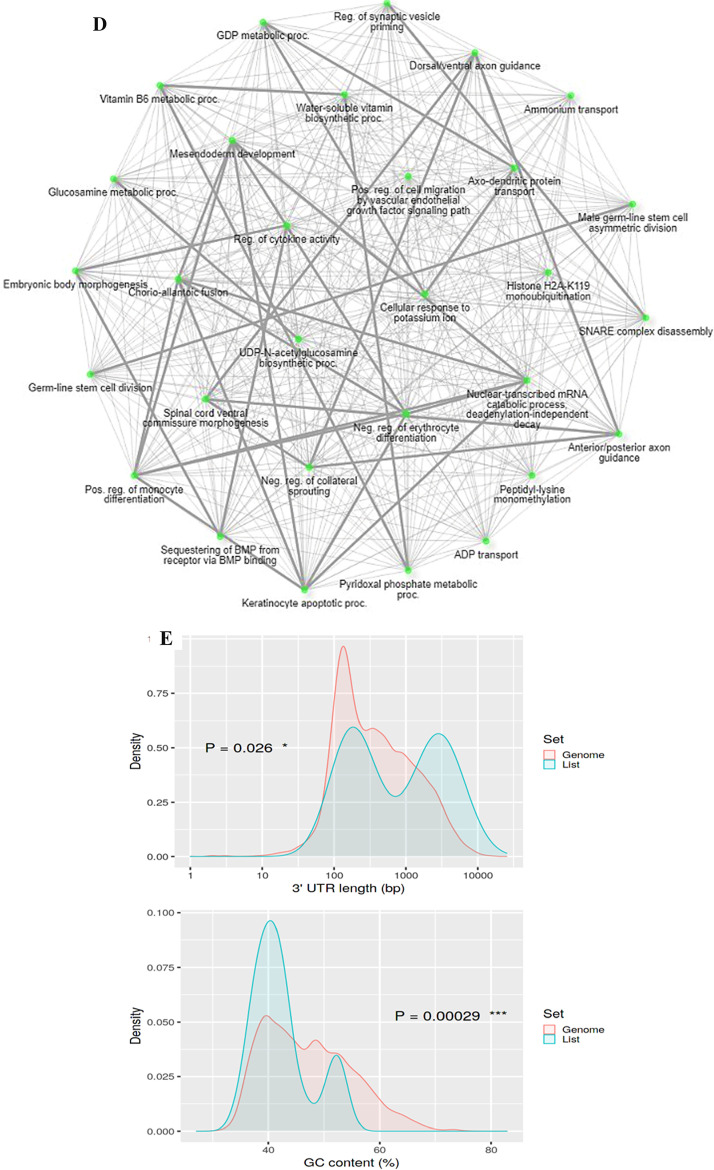
Functional annotation of targeted genes of *C. longa* using Gene Ontology. List of targets that have been thoroughly functionally analysed in terms of **A**. biological processes, **B**. molecular functions, **C**. cellular components. (The chart displays the functional hits as well as the fold enrichment of genes), **D**. Enriched GO biological component terms visualized as a network. Darker nodes are more significantly enriched gene sets. Bigger nodes represent larger gene sets. Thicker edges represent more overlapped genes and **E**. Distribution of the lengths of 3’ UTRs in query genes versus other coding genes in the genome and % of GC content for target genes. Colour red shows high fold enrichment and blue low fold enrichment with an FDR limit of < 0.05

Gene ontology research of *C. longa* miRNAs targets revealed diverse roles, including genes involved in transcriptional regulators, and pathway involvement in signalling and metabolic processes. Various enrichment pathways, molecular activities, biological processes, and cellular components associated to human target genes are detected by the ShinyGO database. ALPK1, ATRN, CYB5B, SETD7, ZFP36L1, ETV5, and SRXN1 were among the genes involved in stress reactions. ALPK1, GAB1, DLG2, GREM2, DCC, ZFP36L1, and ETV5 were discovered to be involved in the regulation of the multicellular developmental process. These target genes are also involved in cell adhesion and the immunological response. CYB5B, GREM2, TNFSF15, and PPP3R2 were shown to be involved in the control of molecular activities. Molecular transducer and oxidoreductase activity for CYB5B, PNPO SRXN1, CLEC2D, DCC, and ATRN were also reported. These genes were found significantly Distribution of the lengths of 3' UTRs in target genes versus other coding genes in the genome. Moreover, these genes also show significant GC content compared with the rest in the genome. The numerous relationships between target genes and associated GO functions are depicted in Fig. 2A–E.

### Gene interaction predictions for targeted genes

**Fig. 3:**
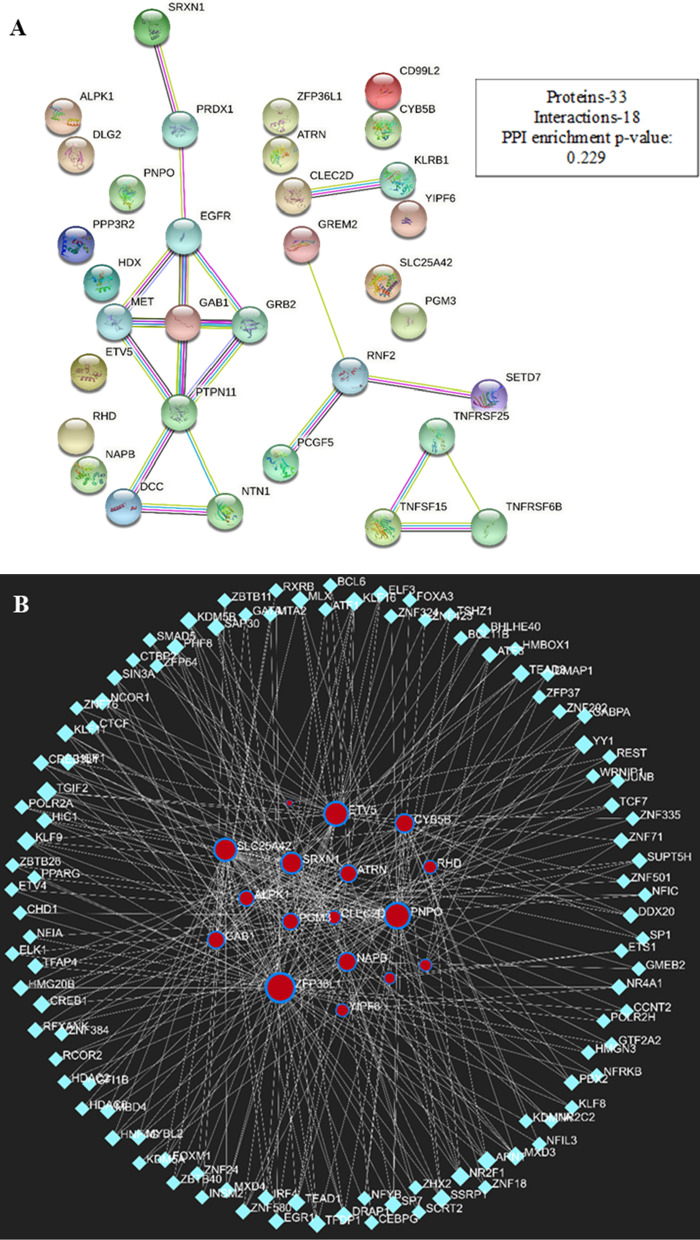
**A** The enrichment analysis of protein- protein annotation of target genes through STRING and **B** Gene–gene interaction of targeted genes network analyst algorithm. Red node shows the predicted human target gene and blue node shows the interaction with target genes

**Fig. 4:**
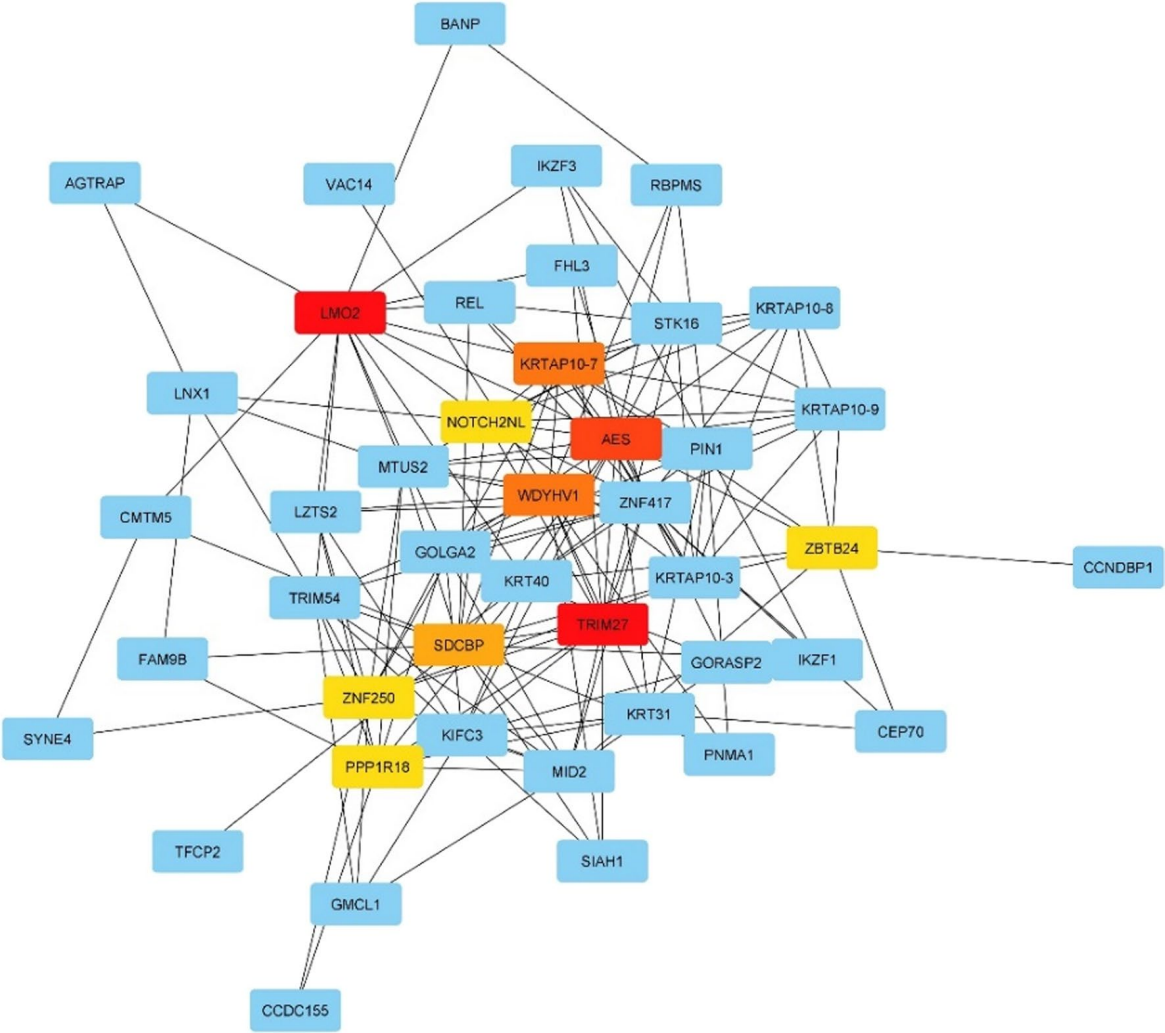
The Cytoscape plugin cytohubba was used to identify the top hub nodes using the bottleneck method. Hub nodes in an enrichment analysis network study of their target genes. A colour-coded systems used to denote them from highly necessary (red) to necessary hub nodes (yellow)

We intended to use STRING and Network analyst databases in our study to identify protein–protein, gene–gene interrelationships and also the KEGG pathway functions for these networks of interacting molecules attributed to our human target gene of *C. longa* miRNAs for linking with cross-kingdom analysis (Fig. 3A, B). Large databases and an online platform are available for the computational analysis of protein–protein linkage. Some well-known in silico tools in use include IMEX CONSORTIUM, UniProt Consortium, BioGRID, HINT, iRefWeb, APID, GeneMANIA, HumanNet, and FunCoup. The prediction of protein–protein interactions yields a wealth of information about functional connectivity, pathways, and high-throughput experimental interactions [18–20]. *C. longa* miRNAs predicted targets interacted with numerous pathways and other gene we can anticipate that single miRNA can regulate multiple gene, gene functions, and gene-associated pathways (Fig. 4). These gene alterations may lead to changes in normal physiology and cause disease which are major cause of cancerous malignancies. Changes in Cytochrome b target gene, which is involved in lipid metabolism and may cause chemical carcinogenesis. On chromosome 18, the DCC gene is located, and its inactivation is linked to the development and metastasis of colorectal cancer [32]. Furthermore, research indicates that upregulating Gab1 increases breast cancer (BCs) and metastasis by separating the PAR complex, which has been identified as a major regulator of EMT. This suggests that Gab1 may serve as a biomarker for BCa that has spread to other organs (Wang et al., 2019). In a study using zi rats to describe the function of the ATRN gene in the central nervous system (CNS), researchers discovered that when ATRN gene function is lost, reactive oxygen species (ROS) are induced, which causes neurodegeneration and demonstrates the importance of ATRN gene accumulation in the CNS [33]. TNFSF15, a member of the tumour necrosis factor ligand superfamily, modulates inflammatory disorders and MAPK/NF-κB/PI3K signalling pathway. Alteration in this pathway leads to many diseases condition including cancer, autoimmune disorders, etc. [34] (Table 3). This analysis concludes that *C. longa* miRNAs, on the other hand, have the ability to regulate transcription and can act as epigenetic modifiers. A single miRNA interacts with a number of genes, while a single gene interacts with numerous miRNAs. As a result, these miRNAs-gene screenplays adapt to control a variety of illnesses and disease regulatory pathways.

**Table 3:** The prediction of protein annotation pathways of a *C. longa* miRNAs targets using the STRING tool

ID	KEGG description	Matching proteins in network
hsa01521	EGFR tyrosine kinase inhibitor resistance	GAB1, EGFR, MET, GRB2
hsa04360	Axon guidance	NTN1, MET, PTPN11, PPP3R2, DCC
hsa05205	Proteoglycans in cancer	GAB1, EGFR, MET, PTPN11, GRB2
hsa05211	Renal cell carcinoma	GAB1, MET, PTPN11, GRB2
hsa04014	Ras signalling pathway	GAB1, EGFR, MET, PTPN11, GRB2
hsa04072	Phospholipase D signalling pathway	GAB1, EGFR, PTPN11, GRB2
hsa05226	Gastric cancer	GAB1, EGFR, MET, GRB2
hsa05225	Hepatocellular carcinoma	GAB1, EGFR, MET, GRB2
hsa05120	Epithelial cell signalling in Helicobacter pylori infection	EGFR, MET, PTPN11
hsa05223	Non-small cell lung cancer	EGFR, MET, GRB2
hsa04012	ErbB signalling pathway	GAB1, EGFR, GRB2
hsa05210	Colorectal cancer	EGFR, GRB2, DCC
hsa05235	PD-L1 expression and PD-1 checkpoint pathway in cancer	EGFR, PTPN11, PPP3R2
hsa05215	Prostate cancer	EGFR, ETV5, GRB2
hsa04722	Neurotrophin signalling pathway	GAB1, PTPN11, GRB2
hsa04650	Natural killer cell-mediated cytotoxicity	PTPN11, PPP3R2, GRB2
hsa04010	MAPK signalling pathway	EGFR, MET, PPP3R2, GRB2
hsa04068	FoxO signalling pathway	SETD7, EGFR, GRB2
hsa04630	JAK-STAT signalling pathway	EGFR, PTPN11, GRB2
hsa05206	MicroRNAs in cancer	EGFR, MET, GRB2
hsa05144	Malaria	KLRB1, MET

#### Hub node identification

**Fig. 5:**
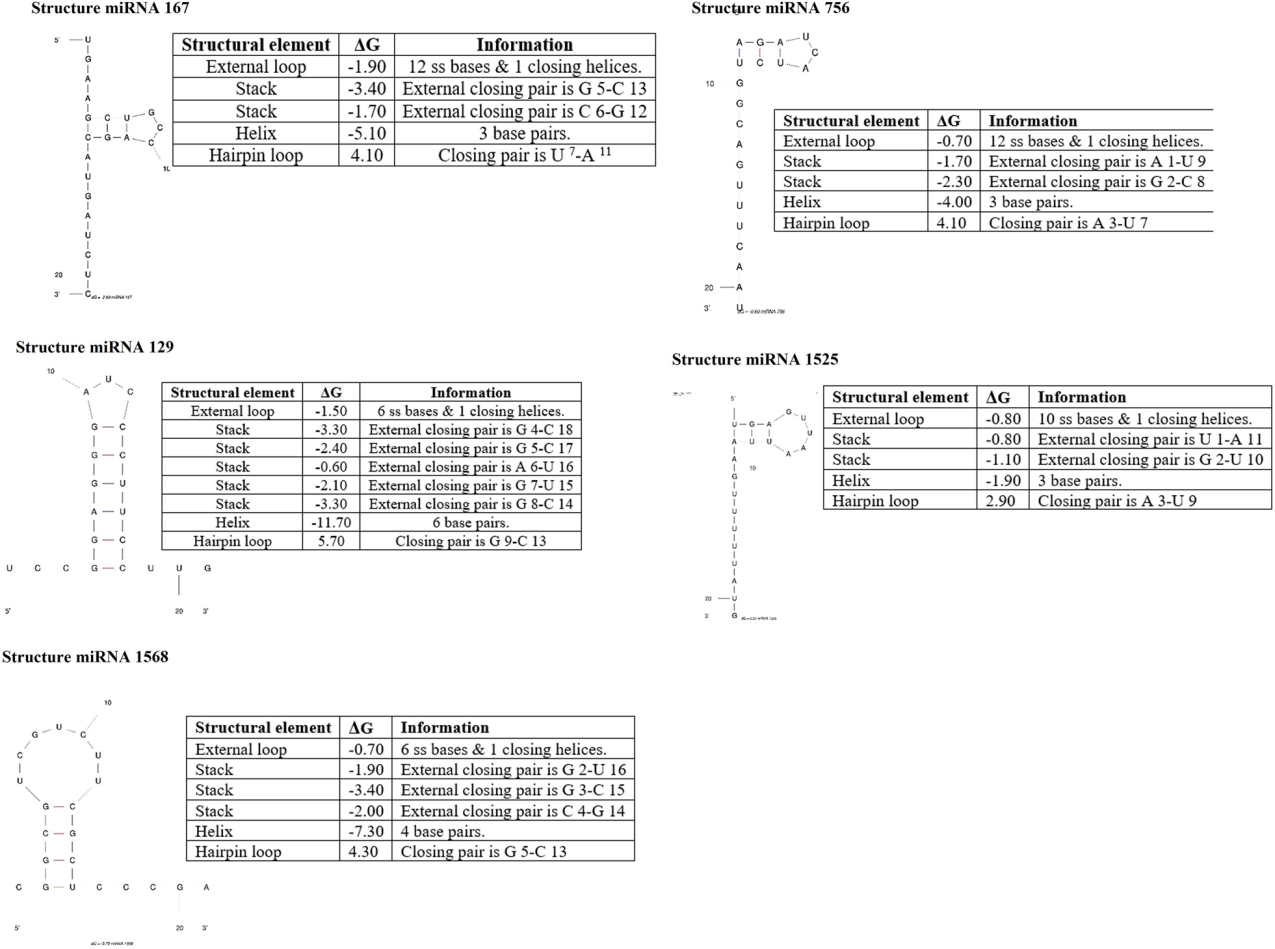
The structure prediction of *C. longa* miRNA by RNACofold

The targeted genes connection was identified hubs with the highest numbers of interactions. Degree method was applied to identify top 30 hub nodes using centrality parameters such as bottleneck, Stress and betweenness concerning significance and biological processes. In the hub interaction score 23 and the highest number of interactions, the topmost hub node was LMO2 (LIM Domain Only 2) key regulator of hematopoietic stem cells and cancer malignancies. Other significant proteins such as TRIM27 (tripartite motif containing 27), AESV (amino-terminal enhancer of split), KRTAP10-7 (Keratin Associated Protein 10-7), WDYHV1(WDYHV motif containing 1), SDCBP (Syndecan Binding Protein), NOTCH2NL (Notch homolog 2 N-terminal-like PROTEIN), ZNF250 (Zinc Finger Protein 250), PPP1R18 (protein phosphatase 1 regulatory subunit 18) and ZBTB24 (Zinc Finger And BTB Domain Containing 24) were identified as the other top nodes with 1,3,4,4,6,7,7,7 and 7 rank, respectively (Fig. 5). This top node indicates the sophisticated network linkages and track down the important role of LMO2 and TRIM27 in the signalling cascade by interacting proteins in this network.

### RNA secondary structure prediction of ***C. longa*** miRNAs for human target genes

In our study, the predicted secondary structure of nine *C. longa* miRNAs was identified. A dot in the dot-bracket plot depicts the unpaired position of two sequences, whereas the bracket represents the matching pair. Furthermore, RNA sequences are in a heterodimer structure with the least amount of free energy and separate the two sequences. According to the sequence structure shown in the table, *C. longa* miRNAs has an energy level of under − 20 kcal/mol, which is favourable for post-transcriptional gene silencing processes [8] (Fig. 4) (Table 4). The secondary structure of *C. longa* miRNAs was also calculated using thermodynamic approaches and Mfold, with the folding formula ΔG = ΔH − TΔS [27] (Table 5).

**Table 4:** The prediction of secondary structure prediction of miRNAs using RNAcofold

miRNAs	miRNAs secondary structure	Dot bracket plot	MFE (kcal/mol)
miRNA 167		UCUAGUACGACCGUCGAAGU&UGAAGCUGCCAGCAUGAUCU ……..((.(((……&….(((…)))))).))	− 2.50
miRNA 129		UUCCUUCCCUAGGGAGGCCU&UCCGGAGGGAUCCCUUCCUU ..((((((…))))))…&…((((((…)))))).	− 14.30
miRNA 756		AACUUUGACGGUCUACUAGA&AGAUCAUCUGGCAGUUUCAA ((((..((.(((((……&))))).))….))))…	− 6.20
miRNA 1525		GUAUUUUUGAAUUAAUUGAGU&UGAGUUAAUUAAGUUUUUAUG ………((((((((….&..))))))))……….	− 4.30
miRNA 1568		GCCCUCGCUUCUGCUGCGGC&CGGCGUCGUCUUCGCUCCCG (((.((((…….)))).&.)))……………	− 9.00

**Table 5:** The results of calculating the minimum free energy for predicted miRNAs using Mfold

miRNAs structure	ΔG (free energy)	ΔH (enthalpy)	ΔS (entropy)	*T*_*m*_ (°C)
miRNA 167	− 2.90 kcal/mol	− 32.40 kcal/mol	− 95.1 cal/K.mol	67.4
miRNA 129	− 7.50 kcal/mol	–	–	–
miRNA 756	− 0.60 kcal/mol	− 26.70 kcal/mol	84.1 cal/K.mol	44.1
miRNA 1525	0.20 kcal/mol	− 15.90 kcal/mol	− 51.9 cal/K.mol	33.1
miRNA 1568	− 3.70 kcal/mol	− 43.00 kcal/mol	− 126.7 cal/K.mol	66.1

Standard errors are roughly ± 5%, ± 10%, ± 11%, and 2–4 °C for free energy, enthalpy, entropy, and *T*_*m*_, respectively

## Discussion

*Curcuma* *longa*'s cross-kingdom significance in humans can be compared by evaluating the effects of different miRNAs from the plant using computational approaches. The therapeutic potential of *C. longa* and its miRNAs in treating a variety of human ailments is determined by this study. *C. longa* is a notable medicinal plant that is frequently used to treat a variety of cellular and pathological diseases. Numerous phytochemical investigations have been run to identify the key pharmacological properties of this therapeutic plant [35]. The basic mechanism, through which medicinal plants alter the human DNA is yet unknown. Most eukaryotic genomes, including those of plants, now express miRNAs as a substantial governing element. We applied an in silico strategy in this study to predict miRNAs from *C. longa* data and to identify their targets in Homo sapiens, which has been made possible by the gene function of these miRNAs. We discovered nine miRNAs by using the data on recognized plant miRNAs that is available in the miRNEST database. The precursor miRNA constructs were examined for other energy factors like MFE and stability as well as their capacity to acquire hairpin loops. It is well known that the miRNA secondary structure is more stable the lower the MFE value [36]. The two main characteristics in this study that were applied in the psRNATarget tool to find possible human targets were cross-kingdom complementarity and target identification. Potential miRNAs targets for *C. longa* were predicted in this analysis. The majority of the anticipated miRNAs targets were transcription factor coding genes. The projected miRNAs' GC content and 3' UTR length were determined to be significant, respectively, at *p*- 0.00029 and *p*- 0.026. Predicted miRNAs had energy criteria that were negative and within the acceptable range for RNA-mediated gene encoding. Our findings show that nine miRNAs sequences, including miRNA1525, miRNA129, miRNA167 (1, 2, 3, and 4), miRNA756, andmiRNA1568, are associated with the transcriptome of the *C. longa*. Evidently, 23 crucial human genes involved in biological, physiological, and metabolic processes are influenced by all of these miRNAs. Four of the 23 target genes were discovered to play a regulatory role in the hallmarks of cancer, including angiogenesis, metastasis, apoptosis, and cell proliferation. Furthermore, it was discovered that several targets, such as DLG2, ETV5, PGM3, and ALPK1, were key regulators of neurological diseases (Table 2). The results of the cross-kingdom investigation show that all 23 *C. longa* predicted genes miRNAs were the primary regulators of human cellular, metabolic, and biological processes, as well as a number of signalling networks. Potential targets LMO2, TRIM27, AES, KRTAP10-7, WDYHV1, SDCBP, NOTCH2NL, ZNF250, PPP1R18 AND ZBTB24 are examples of genes regulated by cross-kingdom research that have been discovered to play a significant role in a number of disorders, including hematopoietic stem cell formation [37], oncogenic roles in various malignancies [38, 39], regulation of NF-κB signalling cascade [40], neurogenesis [41] and immunodeficiency [42]. These point to the potential anti-carcinomous properties of these miRNAs for disease prevention and therapeutic importance. The results accomplish that using bioinformatics resources, *C. longa* miRNAs and their predicted human target genes were identified. In upcoming cross-kingdom research, these anticipated miRNAs might be helpful in addressing the disease-centric investigations. These miRNAs from *C. longa* have potential as therapeutic markers in the interdisciplinary diagnosis of diseases that are associated to genes. These miRNAs will be crucial markers for the diagnosis, prognosis, and treatment of a wide spectrum of disorders soon. However, the potential utility of these identified miRNAs further validated through *in vitro* and *in vivo* studies are required for these *in silico* findings. This constructive approach may potentially prove useful in future inter-kingdom study on disease analysis.

## Conclusions

Our findings reveal that, *C. longa* miRNAs have immune-modulatory, DNA-repairing, anti-tumour, antiviral, anti-inflammatory, and anti-oxidative properties. These characteristics are identical to those described for *C. longa* active phytochemicals. As a result, part of *C. longa*'s health advantages may be mediated by its miRNAs. The outcomes of this study could help researchers better understand how dietary miRNAs affect consumer physiology across kingdoms. This research on *C. longa* miRNAs provides insight on the development of short RNA biomarker treatments for target prediction and identification for a variety of diseases that are riding the wave of new innovations in plant sciences.

## Data Availability

On request, access to data will be available.

## Abbreviations

SARS-CoV-2: Severe acute respiratory syndrome coronavirus-2
Mpro: Main protease
*C. longa*: *Curcuma Longa*
AIDS: Human immunodeficiency virus infection and acquired immune deficiency syndrome
RNAi: RNA interference
miRNA: Micro RNA
siRNAs: Small-interfering RNAs
ORF: Open reading frame
GNA12: G-protein subunit alpha 12
HEK293: Human embryo kidney cells
2-OH group: 2'-Hydroxyl group
A. thaliana: Arabidopsis thaliana
MAPK: Mitogen-activated protein kinases
BCs: Breast cancer
NF-B: Nuclear factor kappa B
CNS: Central nervous system
ROS: Reactive oxygen species
MFE: Minimum folding energy
NMR: Nuclear magnetic resonance
